# An engineered bacteria-chitosan hybrid robot for lactate depletion and synergistic STING activation to potentiate bladder cancer immunotherapy

**DOI:** 10.1016/j.mtbio.2026.103593

**Published:** 2026-08-25

**Authors:** Baodan Zhang, Jie Zhu, Zhenyu Hong, Yuhang Wang, Yunong Wang, Fangdie Ye, Liping Tao, Yongyong Lu

**Affiliations:** a Department of Urology, The First Affiliated Hospital of Wenzhou Medical University, Wenzhou, Zhejiang, 325000, China; b Department of Psychiatry, The Second Affiliated Hospital and Yuying Children's Hospital of Wenzhou Medical University, Wenzhou, Zhejiang, 325000, China; c Fudan Institute of Urology, Huashan Hospital, Fudan University, Shanghai, 200000, China; d Department of Gastroenterology, The First Affiliated Hospital of Wenzhou Medical University, Wenzhou, Zhejiang, 325000, China; e Institute of Urology, Wenzhou Medical University, Wenzhou, 325000, China

**Keywords:** Bladder cancer, Immunotherapy, STING activation, Chitosan nanoparticle, Hybrid robot

## Abstract

The treatment of bladder cancer faces multiple challenges, including poor tumor tissue penetration, low drug delivery efficiency, and an immunosuppressive microenvironment. To overcome these bottlenecks, this study constructed a novel bacteria-chitosan hybrid robot. The system utilizes electrostatic interactions to combine engineered *Escherichia coli* expressing lactate oxidase with positively charged chitosan nanoparticles, with the latter co-loaded with a chemotherapeutic drug Doxorubicin (DOX) and the DNA repair inhibitor olaparib. Following intravesical instillation, the engineered bacteria actively target and penetrate tumor tissues, significantly enhancing the accumulation and retention of nanomedicine at the lesion site. The bacteria colonizing the tumor continuously metabolize local lactate, which not only enhances chemosensitivity but also effectively alleviates tumor immunosuppression. Importantly, lactate consumption itself directly promotes the activation of the STING pathway. Simultaneously, the DOX and olaparib released synergistically induce DNA damage and cascade-activate the STING pathway. Chitosan itself also enhances STING signaling, thereby synergizing with the metabolic modulation by the engineered bacteria to further stimulate a potent antitumor immune response. Experimental results demonstrate that this hybrid robot can remodel the tumor immune microenvironment through multiple pathways and significantly enhance the therapeutic efficacy of anti-PD-L1 antibody therapy. This study provides a novel delivery strategy of EcLOX-CS@DOX-OLA with active targeting, metabolic modulation, and immune-activating functions for the combined treatment of bladder cancer.

## Graphical abstract

**Figure:**
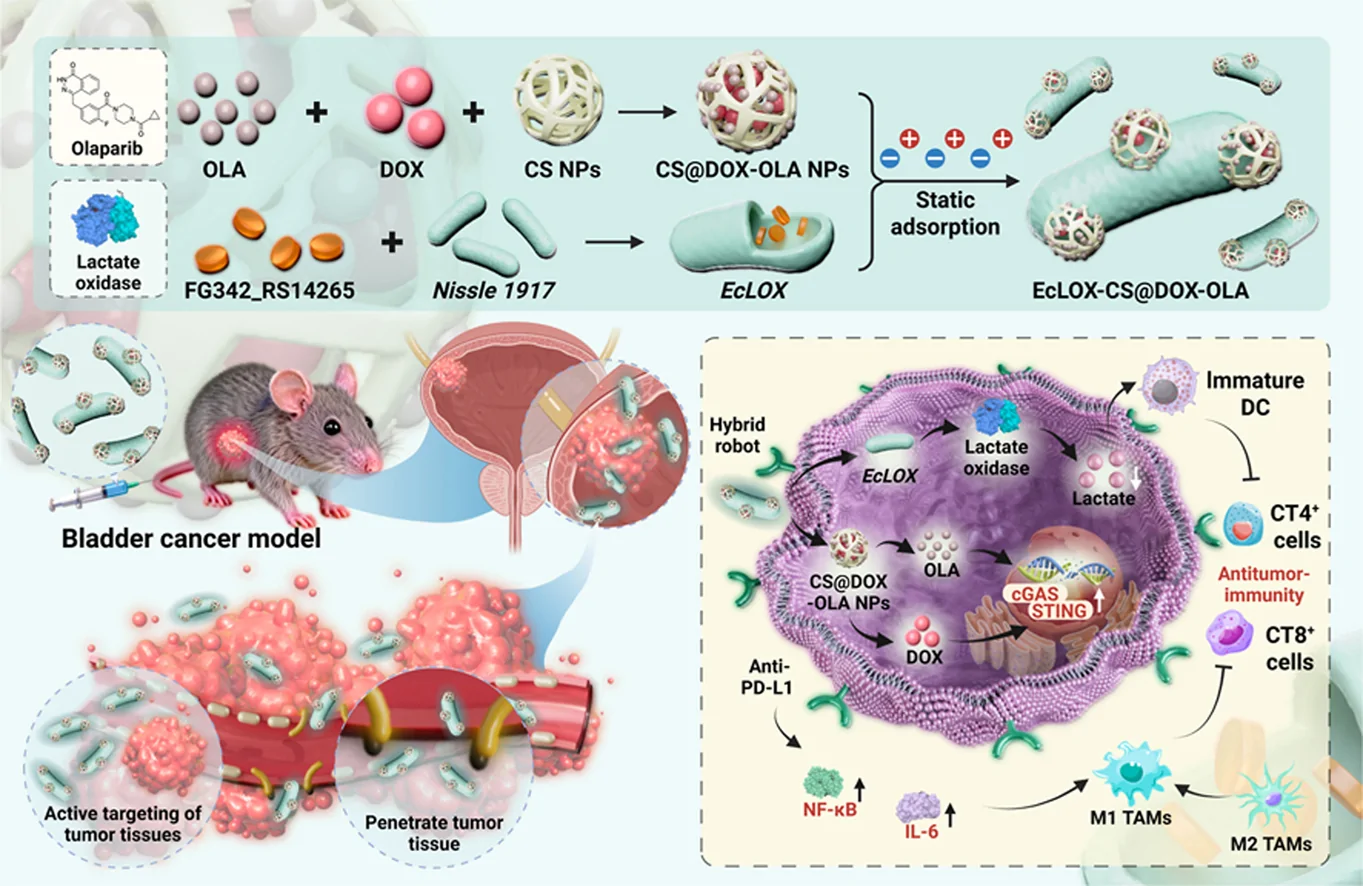

## 1 Introduction

Bladder cancer is one of the most common malignant tumors of the urinary system worldwide, with persistently high incidence and mortality rates posing a serious threat to public health [1]. Current clinical management strategies for bladder cancer are markedly stage-dependent: for non-muscle-invasive bladder cancer, transurethral resection of bladder tumor combined with Bacillus Calmette-Guérin (BCG) intravesical instillation is the standard treatment, yet the recurrence rate remains high; for muscle-invasive or metastatic bladder cancer, systemic chemotherapy remains the cornerstone of therapy [2]. In recent years, with a deeper understanding of the mechanisms of chemotherapy, traditional chemotherapeutic agents, such as cisplatin and doxorubicin are no longer viewed solely for their cytotoxic effects [[3], [4], [5]]. Studies have revealed that these drugs can induce immunogenic cell death in tumor cells, release damage-associated molecular patterns, and enhance anti-tumor immune responses by activating the cGAS-STING pathway [6,7]. This discovery provides a solid theoretical foundation for the synergistic application of chemotherapy and immune checkpoint inhibitors, particularly PD-1/PD-L1 blockers [8]. In recent years, innovative strategies have emerged to enhance STING activation and remodel the immunosuppressive tumor microenvironment. Biomineralized engineered bacterial outer membrane vesicles (OMVs) have been developed to synergistically activate the cGAS-STING pathway and modulate lactate metabolism, demonstrating potent antitumor efficacy [9]. However, despite the theoretical synergistic potential of combining chemotherapy with immunotherapy, the overall response rate in clinical practice for bladder cancer remains suboptimal.

The challenges in bladder cancer treatment are characterized by multi-level and multi-dimensional complexity. At the most fundamental physical and pharmacokinetic level, the unique physiological structure of the bladder and the dense tumor stroma constitute the first barrier. The mucosal layer and tight junctions on the bladder epithelial surface restrict effective drug penetration, while high interstitial fluid pressure and abnormal vascular structures within the tumor further hinder deep drug delivery and uniform distribution, leading to insufficient and heterogeneous intratumoral drug concentrations [10,11]. At the more complex tumor microenvironment level, bladder cancer typically exhibits significant metabolic abnormalities, namely the Warburg effect, resulting in substantial lactate accumulation [12]. This acidic microenvironment not only directly suppresses the activity and proliferation of cytotoxic T cells, promotes the accumulation of regulatory T cells and myeloid-derived suppressor cells, but also induces the polarization of tumor-associated macrophages toward the M2 phenotype, collectively fostering a profoundly immunosuppressive state [13]. Moreover, the acidic pH can affect the stability and activity of certain chemotherapeutic drugs, reducing their therapeutic efficacy. At the most fundamental cellular and molecular mechanism level, tumor cells develop therapeutic resistance through multiple pathways. On one hand, the hyperactivation of DNA damage repair systems, particularly homologous recombination repair and base excision repair pathways, enables tumor cells to rapidly repair DNA damage caused by chemotherapy, diminishing its cytotoxic effects. On the other hand, tumor cells can evade immune surveillance by downregulating MHC class I molecule expression and reducing tumor antigen release [14]. Even if the STING pathway is partially activated, it often fails to elicit a robust and durable adaptive immune response. These barriers do not exist in isolation but are interwoven and mutually reinforcing, forming a highly complex and dynamically evolving resistance network.

Confronted with these multi-layered challenges, conventional monotherapies or simple drug combinations struggle to achieve breakthroughs. In recent years, with the convergence of synthetic biology, nanotechnology, and immunology, the design of bio-abiotic hybrid systems capable of active targeting, intelligent responsiveness, and multi-mechanistic synergy has emerged as a research frontier [15]. For example, engineered probiotics have been developed to achieve tumor-targeted radio-sensitization and immune activation through hypoxia-directed delivery and surface modification with nanomaterials [16], highlighting the versatility of living bacteria as therapeutic platforms. While engineered probiotics have been designed to achieve tumor-targeted radio-sensitization and immune activation via hypoxia-directed delivery [17]. Based on this concept, this study constructed a bacteria-chitosan hybrid robot. This system is synergistically designed across three dimensions: First, genetically engineered *Escherichia coli* serves as an active delivery vehicle, leveraging its natural tropism and motility to overcome physical barriers and achieve deep tumor targeting and colonization. These engineered bacteria continuously express lactate oxidase, converting lactate in the tumors, thereby reversing immunosuppression at its source. Second, positively charged chitosan nanoparticles constructed via electrostatic assembly can co-encapsulate traditional chemotherapeutic drugs (to induce immunogenic cell death) and the PARP inhibitor olaparib (to block DNA damage repair). Their synergy not only enhances direct cytotoxic effects but also increases the accumulation of cytoplasmic DNA, providing ample ligands for cGAS-STING pathway activation. Notably, the chitosan carrier itself has been demonstrated to act as an effective STING agonist, further amplifying the intensity and persistence of innate immune pathway activation. Finally, lactate consumption by the engineered bacteria and STING activation by the nanoparticles form a positive feedback loop: lactate clearance improves immune cell function and enhances downstream effects of the STING pathway, while STING activation promotes immune cell infiltration and activation, boosting tumor surveillance and clearance. This multi-level, multi-mechanistic synergistic action aims to systematically remodel the tumor immune microenvironment, transforming “cold” tumors into “hot” ones, thereby creating more favorable conditions for response to PD-L1 blockade therapy. This study not only provides a novel technological platform for bladder cancer treatment, but also offers fresh perspectives on understanding the significance of metabolic-immune interactions in cancer therapy, potentially advancing bladder cancer combination strategies toward more precise and effective directions.

## 2 Results

### 2.1 Preparation and characterization of CS@DOX-OLA nanoparticles

**Fig. 1:**
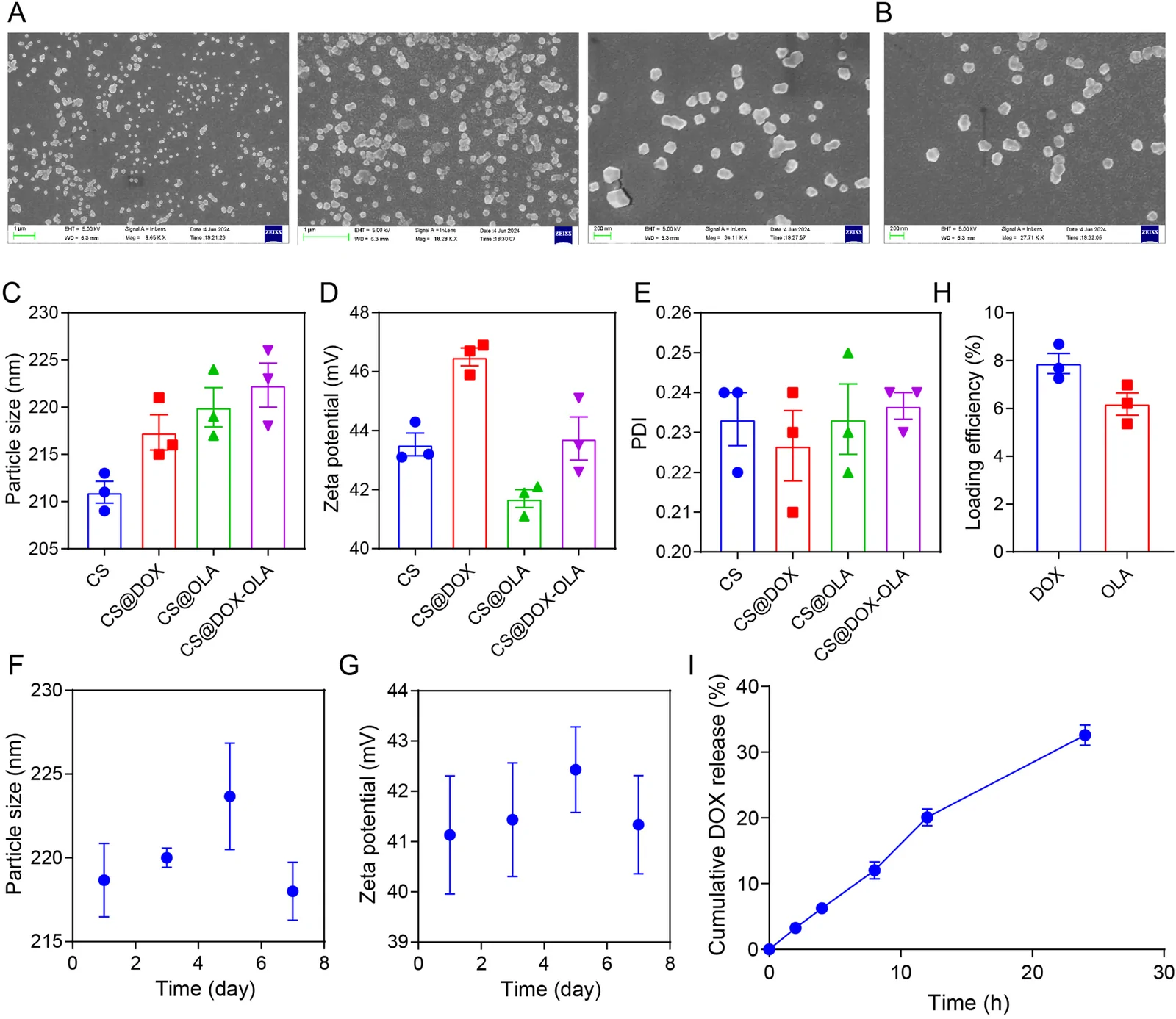
**Preparation and characterization of CS nanomedicine**. (a) SEM of CS nanoparticles with different magnifications. (b) SEM of CS@DOX-OLA nanoparticles. (c) Particle size of different CS nanoparticles (n = 3). (d) Zeta potential of different CS nanoparticles (n = 3). (e) PDI of different CS nanoparticles (n = 3). (f) Particle size stability of CS@DOX-OLA nanoparticles (n = 3). (g) Zeta potential stability of CS@DOX-OLA nanoparticles (n = 3). (h) DOX and OLA loading efficiency of CS@DOX-OLA nanoparticles (n = 3). (i) Cumulative release of DOX of CS@DOX-OLA nanoparticles (n = 3).

To enhance the immunostimulatory effect of chemotherapeutic drugs, we constructed chitosan nanoparticles co-loaded with the chemotherapeutic agent doxorubicin and the PARP inhibitor olaparib. Olaparib amplifies doxorubicin-induced STING activation in tumors by blocking DNA damage repair. Moreover, chitosan has also been shown to effectively enhance STING pathway activation. Scanning electron microscopy images clearly demonstrated that the chitosan nanoparticles exhibited good dispersibility at different magnification levels (Fig. 1A and Fig. S1). For the dual-drug-loaded nanoparticles (CS@DOX-OLA), the surface morphology and dispersibility remained unaffected (Fig. 1B). The size, zeta potential, and polydispersity index of various nanoparticles were monitored by dynamic light scattering. Compared with blank chitosan nanoparticles, the drug-loaded CS nanoparticles showed a slight increase in size (Fig. 1C), while the zeta potential remained largely unchanged—both maintaining a positive surface charge (Fig. 1D). Correspondingly, the PDI of CS nanoparticles before and after drug loading showed little variation, remaining around 0.25, which indicates favorable dispersion stability (Fig. 1E). After storing CS@DOX-OLA nanoparticles in PBS or urine medium for one week, both particle size and surface potential as well as PDI changed minimally, confirming their good stability and suitability for biological applications (Fig. 1F and G, and Fig S2). Considering that intravesical instillation directly exposes the nanoparticles to urine, we further evaluated their colloidal stability in artificial urine over 7 days. As shown in Fig. S3, CS@DOX-OLA nanoparticles maintained stable particle size and zeta potential in urine. To better simulate the weakly acidic bladder tumor microenvironment, we further evaluated the drug release profile at pH 6.5. As shown in Fig. S4, CS@DOX-OLA nanoparticles exhibited accelerated drug release at pH 6.5 compared to pH 7.4, with approximately 75% DOX and 65% OLA released within 24 h, confirming a pH-responsive release behavior that favors drug liberation within the acidic tumor microenvironment.

CS@DOX-OLA nanoparticles effectively co-encapsulated the two drugs, with a drug loading capacity of approximately 8% for doxorubicin and about 6% for olaparib (Fig. 1H). Furthermore, after one week of storage, the drug loading rates showed no significant change, indicating minimal drug leakage from the chitosan nanoparticles during preservation (Fig. S5). Finally, we examined the release profiles of both drugs from CS@DOX-OLA nanoparticles. Over the 24-h monitoring period, both drugs were released gradually (Fig. 1I and Fig. S6). This sustained release behavior is expected to contribute to improved antitumor efficacy.

### 2.2 Preparation and characterization of EcLOX-CS

**Fig. 2:**
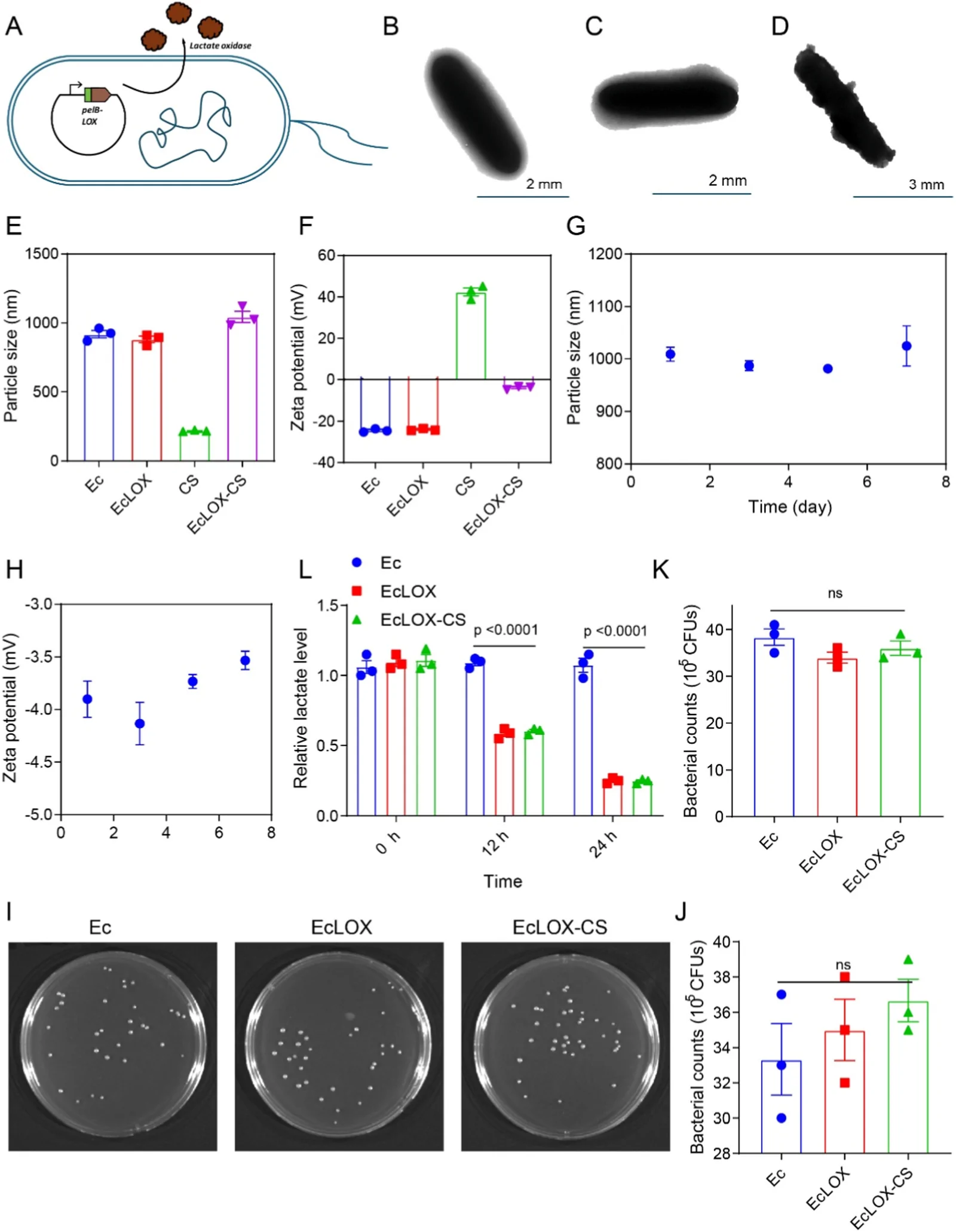
**Preparation and characterization of EcLOX-CS**. (a) Schematic illustration of *E. coli* expressing lactate oxidase. (b) TEM of wild-type *E. coli* (Ec). (c) TEM of EcLOX. (d) TEM of EcLOX-CS. (e) Particle size of different particles (n = 3). (f) Zeta potential of different particles (n = 3). (g) Particle size stability of EcLOX-CS (n = 3). (h) Zeta potential stability of EcLOX-CS (n = 3). (i) Colony photographs of different bacteria onto LB agar plates. (j) Quantitative analysis of different bacteria onto LB agar plates (n = 3). (k) Quantitative analysis of different bacteria onto LB agar plates after one-week storage (n = 3). (l) Relative lactate consumption in a lactate-added medium after treatment with different bacteria (n = 3). One-way Anova analysis; ns, no significant.

To enhance the tumor targeting and penetration ability of CS@DOX-OLA nanoparticles, we constructed a bacteria-chitosan hybrid robot. As living entities, bacteria can specifically target hypoxic regions of tumors and enhance penetration into tumor tissues. Moreover, bacteria serve as excellent chassis for genetic engineering, enabling the introduction of specific functions through synthetic biology. This means that engineered bacteria can not only act as drug carriers, but also be genetically modified to exert specific effects on tumors. Therefore, to improve the tumor delivery and penetration of CS@DOX-OLA and to enhance the antitumor efficacy of STING activation induced by CS@DOX-OLA, we engineered *Escherichia coli* to express lactate oxidase (EcLOX) (Fig. 2A). This modification allows continuous depletion of immunosuppressive lactate as the bacteria proliferate within tumors. By electrostatic self-assembly, positively charged CS@DOX-OLA nanoparticles and negatively charged engineered bacteria were assembled to form EcLOX-CS hybrids. Transmission electron microscopy imaging showed that EcLOX exhibited no obvious morphological change compared to wild-type *E. coli (Ec)*, both displaying smooth rod-shaped structures (Fig. 2B and C). In contrast, EcLOX-CS hybrids showed numerous nanoparticles attached on their surface, confirming the successful modification with CS@DOX-OLA nanoparticles (Fig. 2D).

Similarly, we characterized the size and surface potential of different bacterial samples using DLS. Compared with *Ec* and engineered EcLOX, the EcLOX-CS hybrid system showed a slight increase in hydrodynamic size, consistent with the surface modification by chitosan nanoparticles (Fig. 2E). Surface potential analysis indicated that both *Ec* and EcLOX exhibited typical negative charges, with zeta potentials around −20 mV and similar to each other. In contrast, the surface potential of EcLOX-CS was significantly higher, approximately −4 mV, which is clearly attributed to the coating with positively charged CS@DOX-OLA nanoparticles (Fig. 2F). Furthermore, during one-week storage in PBS and urine medium, neither the size nor the surface potential of EcLOX-CS changed significantly (Fig. 2G and H), indicating good colloidal stability of the bacteria-nanoparticle hybrid system. Given that intravesical instillation exposes the hybrid to a complex urine environment, we further evaluated its stability in simulated urine medium. As shown in Fig. S7A-D, EcLOX-CS maintained stable particle size and zeta potential over 7 days, retained high bacterial colony counts comparable to those in PBS, and exhibited minimal DOX leakage (less than 8%) after 12 h of incubation. These results collectively confirm that the EcLOX-CS composite can preserve its structural integrity under urine-mimicking conditions, supporting its feasibility for intravesical administration.

Next, we systematically evaluated the proliferative viability of different bacterial strains by plate colony counting. The results showed that the number of colonies formed by the EcLOX-CS hybrid system on LB solid medium was not significantly different from that of Ec or engineered EcLOX, indicating that the CS@DOX-OLA nanoparticle modification did not noticeably affect the basic viability of the bacteria (Fig. 2I and J). Moreover, after one week of storage, the viable counts of all strains showed no significant decrease, further demonstrating the good viability retention of the hybrid system during storage (Fig. S8). To further verify the functionality of the engineered bacteria, we inoculated different strains into lactate-containing medium and measured the residual lactate concentration at various time points. The results demonstrated that both lactate-oxidase-expressing strains, EcLOX and EcLOX-CS, efficiently consumed lactate, with significantly higher consumption efficiency than the wild-type strain, and this consumption exhibited a clear time-dependent manner (Fig. 2K–L, and **S9**). These results confirm that the genetic engineering conferred sustained lactate-metabolizing capability to the bacteria, and this functionality was preserved after nanoparticle modification.

### 2.3 EcLOX-CS@DOX-OLA induces mitochondrial dysfunction and DNA damage to activate the cGAS-STING pathway *in vitro*

**Fig. 3:**
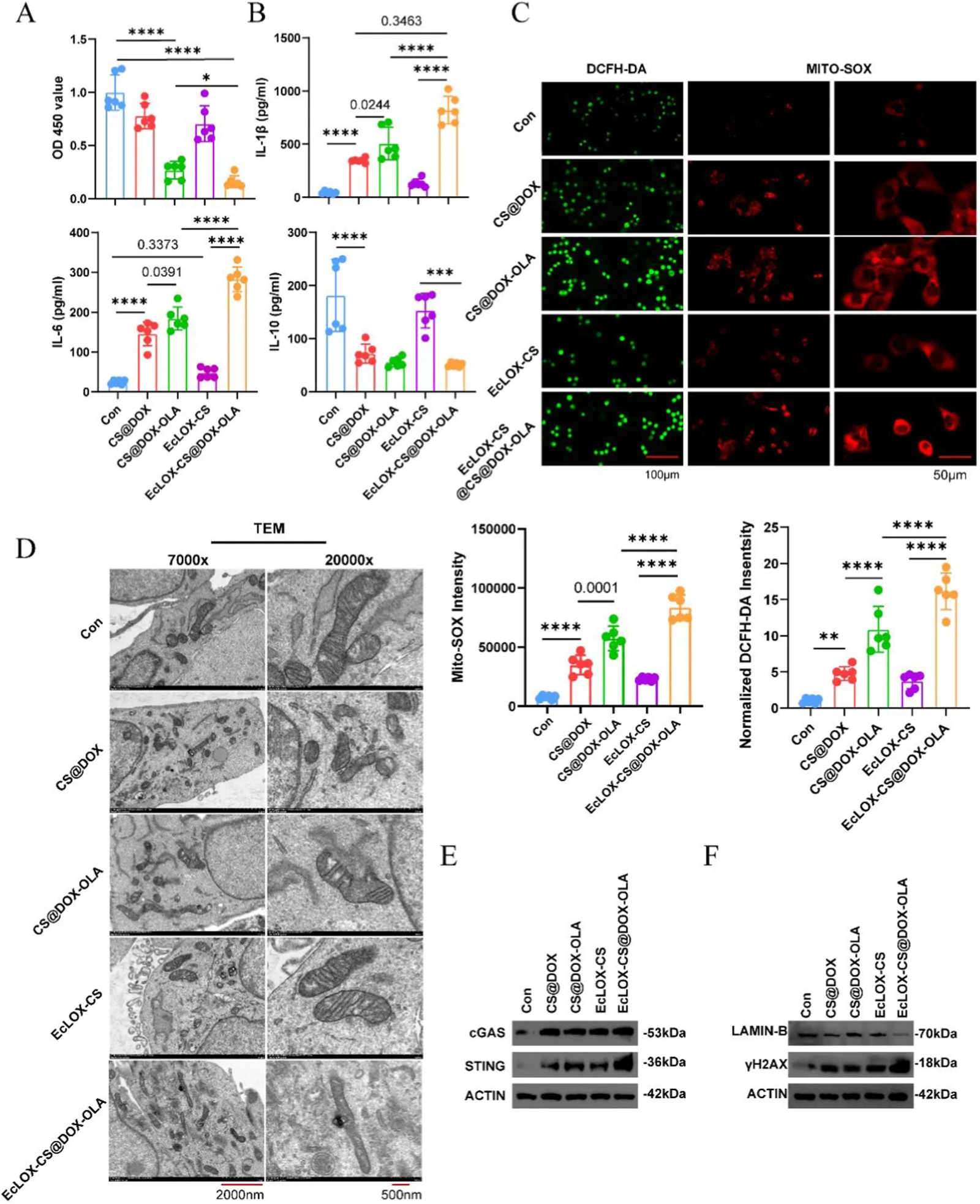
EcLOX-CS@DOX-OLA induces mitochondrial dysfunction, DNA damage, and cGAS-STING activation in bladder cancer cells *in vitro*. (a) Cell viability assessed by CCK-8 assay after treatment with various formulations (n = 6). (b) Secretion of cytokines (IL-1β, IL-6, IL-10) measured by ELISA. (c) Intracellular and mitochondrial ROS levels detected by DCFH-DA and Mito-SOX probes (n = 6). (d) Representative TEM images showing mitochondrial damage. (e) Western blot analysis of key proteins in the cGAS-STING pathway. (f) Western blot analysis of nuclear envelope protein Lamin B1 and DNA damage marker γ-H2AX. One-way Anova analysis; ***p < 0.001, ****p < 0.0001.

We first assessed the cytotoxic and metabolic effects of our various formulations on bladder cancer cells *in vitro*. Compared to the control, all treatment groups exhibited a dose-dependent inhibition of cell viability in CCK-8 assays, with the EcLOX-CS@DOX-OLA hybrids showing the most potent effect (Fig. 3A). To evaluate the impact regarding cytokine secretion, we observed that cancer cells treated with CS@DOX-OLA and EcLOX-CS@DOX-OLA potently induced higher production of IL-1β, IL-6 and less production of IL-10 (Fig. 3B). The final regimen of EcLOX-CS@DOX-OLA induced highest levels of cell damage, evidenced by highest signal intensity of DCFH-DA and Mito-SOX probes (Fig. 3C). Moreover, we observed a marked loss of mitochondrial crista and rupture of mitochondrial membrane (Fig. 3D). Western blot analysis of treated tumor cells confirmed the immunomodulatory mechanism. Treatment with CS@DOX-OLA and EcLOX-CS@DOX-OLA significantly upregulated the expression of key STING pathway proteins (Fig. 3E). To further dissect the individual contribution of lactate depletion to STING activation, Western blot analysis of cGAS and STING total protein expression revealed a stepwise increase across the groups. Notably, EcLOX + blank CS (lactate depletion alone) significantly upregulated both cGAS and STING compared to the PBS control, confirming that lactate depletion per se is sufficient to activate the STING pathway. Meanwhile, Ec + CS@DOX-OLA showed levels comparable to CS@DOX-OLA alone, ruling out non-specific bacterial immune stimulation as a confounding factor (Fig. S10).

Furthermore, these groups showed a decrease in the nuclear envelope protein Lamin B1, alongside an increase in the DNA damage marker (γ-H2AX), collectively proving that the treatment induced DNA damage and cytoplasmic DNA accumulation, which are primary activators of the cGAS-STING pathway (Fig. 3F). The EcLOX-CS@DOX-OLA hybrid robot demonstrated superior *in vitro* cytotoxicity against bladder cancer cells by inducing mitochondrial damage and oxidative stress, triggering DNA damage and cytoplasmic DNA accumulation that activated the cGAS-STING pathway.

### 2.4 Inhibition of DNA repair potentiates STING-dependent apoptosis and macrophage activation

**Fig. 4:**
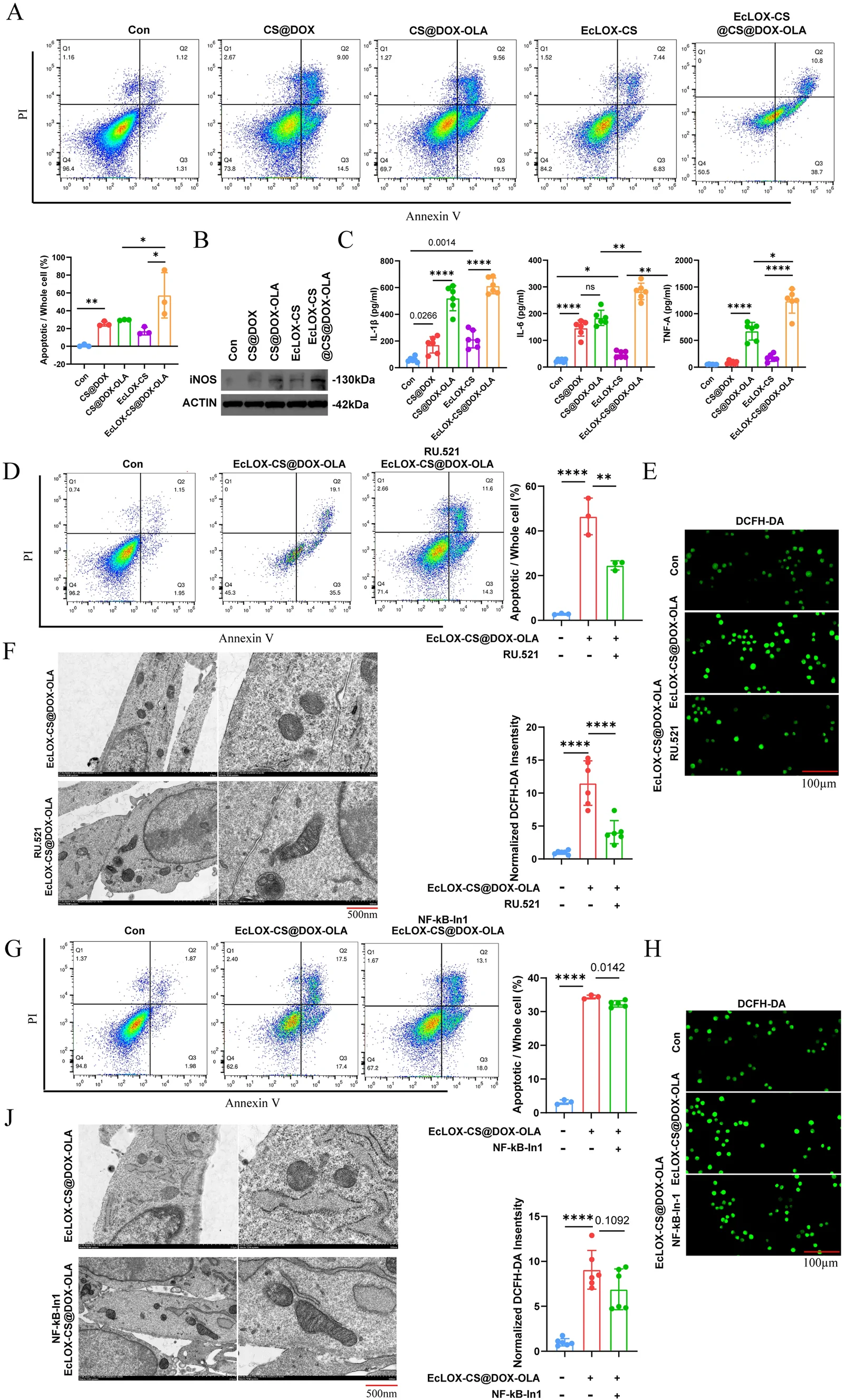
**Inhibition of DNA repair by olaparib potentiates STING-dependent apoptosis and macrophage activation**. (a) Apoptosis of tumor cells assessed by annexin V/PI flow cytometry (n = 3). (b) Western blot analysis of inducible iNOS in macrophages after co-culture with treated tumor cell media. (c) Pro-inflammatory cytokine (IL-1β, IL-6) secretion from co-cultured macrophages measured by ELISA (n = 6). (d-e) Cell viability (n = 3) (d) and intracellular ROS levels (n = 6) (e) upon treatment with the cGAS inhibitor RU521. (f) TEM images of mitochondrial morphology after RU521 co-treatment. (g-h) Cell death (n = 3) (g)and intracellular ROS levels (n = 6) (h) upon inhibition of the downstream NF-κB pathway. (j) TEM images of mitochondrial morphology after NF-κB inhibition. One-way Anova analysis; *p < 0.05, **p < 0.01, ****p < 0.0001.

We confirmed through Annexin V/PI flow cytometry that the combination of DOX and the PARP inhibitor OLA in CS@DOX-OLA and EcLOX-CS@DOX-OLA significantly enhanced tumor cell apoptosis compared to DOX alone, demonstrating that inhibiting DNA repair potentiates direct cytotoxic killing (Fig. 4A). To further characterize the ICD induced by the nanocarrier system, we measured the release of ATP and HMGB1 from treated tumor cells. As shown in Fig. S11, CS@DOX-OLA and EcLOX-CS@DOX-OLA significantly increased extracellular ATP and HMGB1 levels compared to free DOX alone, confirming that the DOX + OLA combination effectively triggers ICD.

In a co-culture system, these formulations also triggered robust macrophage activation. Western blot (Fig. 4B) and ELISA analysis (Fig. 4C) confirmed that CS@DOX-OLA and EcLOX-CS@DOX-OLA treated cell media led to the highest upregulation of macrophage inflammatory marker of iNOS and pro-inflammatory cytokines. To definitively link this activation to the STING pathway, we used the cGAS inhibitor RU.521. RU.521 treatment of cells exposed to EcLOX-CS@DOX-OLA significantly reduced both cell death (Fig. 4D) and intra-cellular ROS accumulation (Fig. 4E), which was sustained by TEM analysis (Fig. 4F). NF-κB is a well-established down-stream signaling pathway regulated by STING activation. Inhibition of the downstream NF-κB pathway also attenuated, but to a lesser extent than RU.521, cell death (Fig. 4G). Through NF-κB inhibition, ROS accumulation was attenuated, however, the statistical analysis revealed no significant differences (Fig. 4H), which was further supplied by TEM analysis (Fig. 4J). The combination of DOX and the PARP inhibitor OLA within the nanocarrier system synergistically enhanced tumor cell apoptosis and triggered robust macrophage activation, a process predominantly mediated through the cGAS-STING pathway.

### 2.5 EcLOX-CS@DOX-OLA achieves potent antitumor efficacy *in vivo* via STING activation

**Fig. 5:**
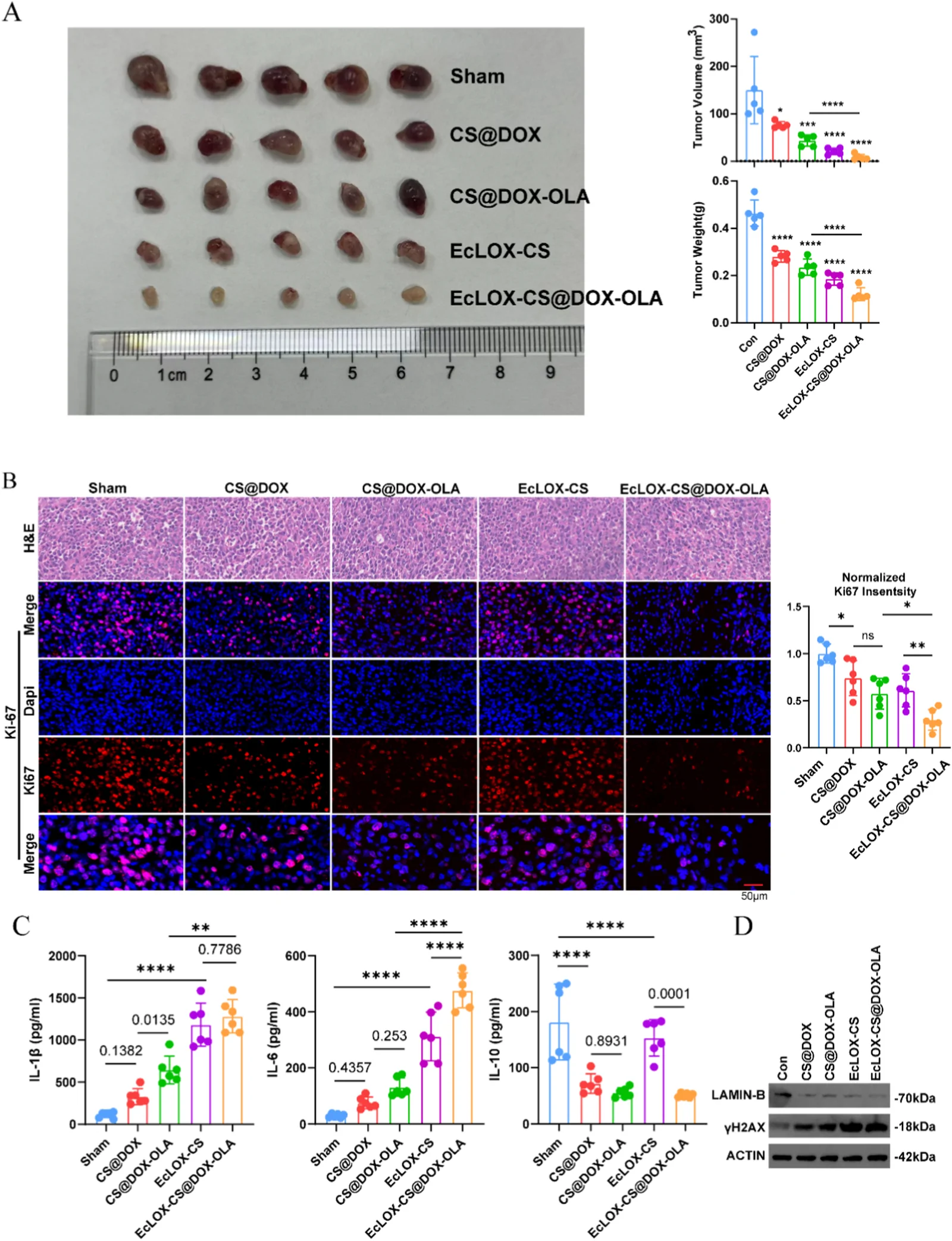
**EcLOX-CS@DOX-OLA achieves potent antitumor efficacy via STING activation in an orthotopic bladder cancer model**. (a) Tumor mass, volume, and representative anatomical images after treatment (n = 5). (b) Tumor proliferation assessed by immunohistochemical staining for Ki67 (n = 6). (c) Serum levels of cytokines (IL-1β, IL-6, IL-10) measured by ELISA (n = 6). (d) Western blot analysis of the DNA damage marker γ-H2AX in tumor tissues. One-way Anova analysis; *p < 0.05, **p < 0.01, ***p < 0.001, ****p < 0.0001.

*In vivo* efficacy was evaluated in an orthotopic bladder cancer model. Mice were treated via intravesical instillation and divided into five groups: PBS (Sham), CS@DOX, EcLOX-CS, CS@DOX-OLA, and EcLOX-CS@DOX-OLA. The EcLOX-CS@DOX-OLA group demonstrated the most potent antitumor activity, showing the lowest tumor mass and volume, and minimal anatomical tumor burden upon dissection (Fig. 5A). Histological analysis corroborated these findings, with the EcLOX-CS@DOX-OLA group showing the most reduced tumor cell proliferation rate. Immunohistochemistry for Ki67 revealed the lowest proliferation rate in this group (Fig. 5B). Serum levels of cytokine were assessed by ELISA analysis, in which EcLOX-CS@DOX-OLA up-regulated IL-1 and IL-6 production, while reduced IL-10 secretion (Fig. 5C). Intra-tumor level of DNA damage was assessed via WB analysis, revealing highest level of DNA damage pattern in the EcLOX-CS@DOX-OLA group (Fig. 5D).

To evaluate the *in vivo* safety of the hybrid system, we performed H&E staining of major organs and measured serum inflammatory cytokine levels. As shown in Fig. S12, no obvious organ damage or systemic inflammation was observed in EcLOX-CS@DOX-OLA-treated mice compared with the control group, confirming the favorable biosafety profile of the hybrid robot.

### 2.6 EcLOX-CS@DOX-OLA reprograms the tumor immune microenvironment by promoting M1 macrophage polarization, STING activation, and T cell infiltration

**Fig. 6:**
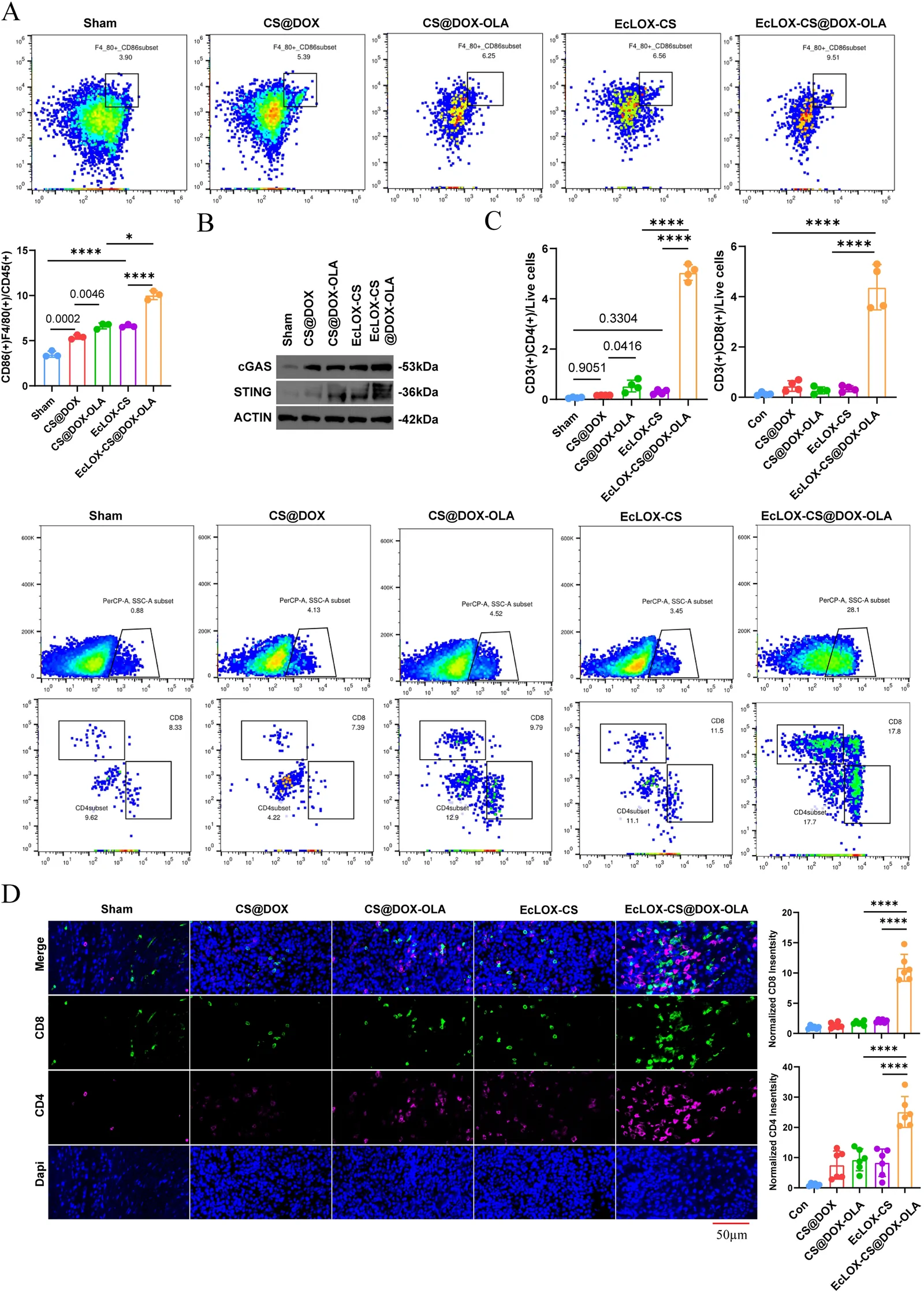
**EcLOX-CS@DOX-OLA reprograms the tumor immune microenvironment *in vivo*.** (a) Flow cytometric analysis of intratumoral M1 macrophage (CD45^+^F4/80^+^CD86^+^) proportions (n = 3). (b) Western blot analysis of tumor STING expression. (c) Flow cytometric analysis of intratumoral CD4^+^ T cell proportions (n = 4). (d) IF analysis of CD4^+^ and CD8^+^ T cell infiltration in tumors (n = 6). One-way Anova analysis; *p < 0.05, ****p < 0.0001.

Various flow cytometry analysis means were applied to assess the intra-tumoral infiltration of different immune cells. EcLOX-CS@DOX-OLA triggers a strong shift towards the inflammatory M1 phenotype, as indicated by the highest CD86 positive and F4/80 positive proportion of the whole CD45 cells (Fig. 6A). To exclude the possibility that the observed antitumor immune response was confounded by non-specific cystitis induced by bacterial instillation, we measured the levels of IL-1β and TNF-α in bladder tissue homogenates. As shown in Fig. S13, no significant elevation of these pro-inflammatory cytokines was detected in the EcLOX or EcLOX-CS@DOX-OLA groups compared to the PBS control, ruling out non-specific bladder inflammation as a contributing factor. Intra-tumor level of cGAS-STING activation was assessed via WB analysis, revealing highest level of STING expression in the EcLOX-CS@DOX-OLA group (Fig. 6B). The proportion of CD4 cells were up-regulated under different CS@DOX treatments (Fig. 6C), while CD8^+^ T cell proportion was only significantly up-regulated under the EcLOX-CS@DOX-OLA treatment, which was also sustained by detailed IF analysis targeting CD4^+^ and CD8^+^ T cells (Fig. 6D). Thus, EcLOX-CS@DOX-OLA potently remodels the tumor immune microenvironment by polarizing macrophages toward the pro-inflammatory M1 phenotype, activating the intratumoral cGAS-STING pathway, and significantly enhancing the infiltration of both CD4^+^ and CD8^+^ T cells.

### 2.7 EcLOX-CS@DOX-OLA synergizes with PD-L1 checkpoint blockade to achieve potent tumor eradication

**Fig. 7:**
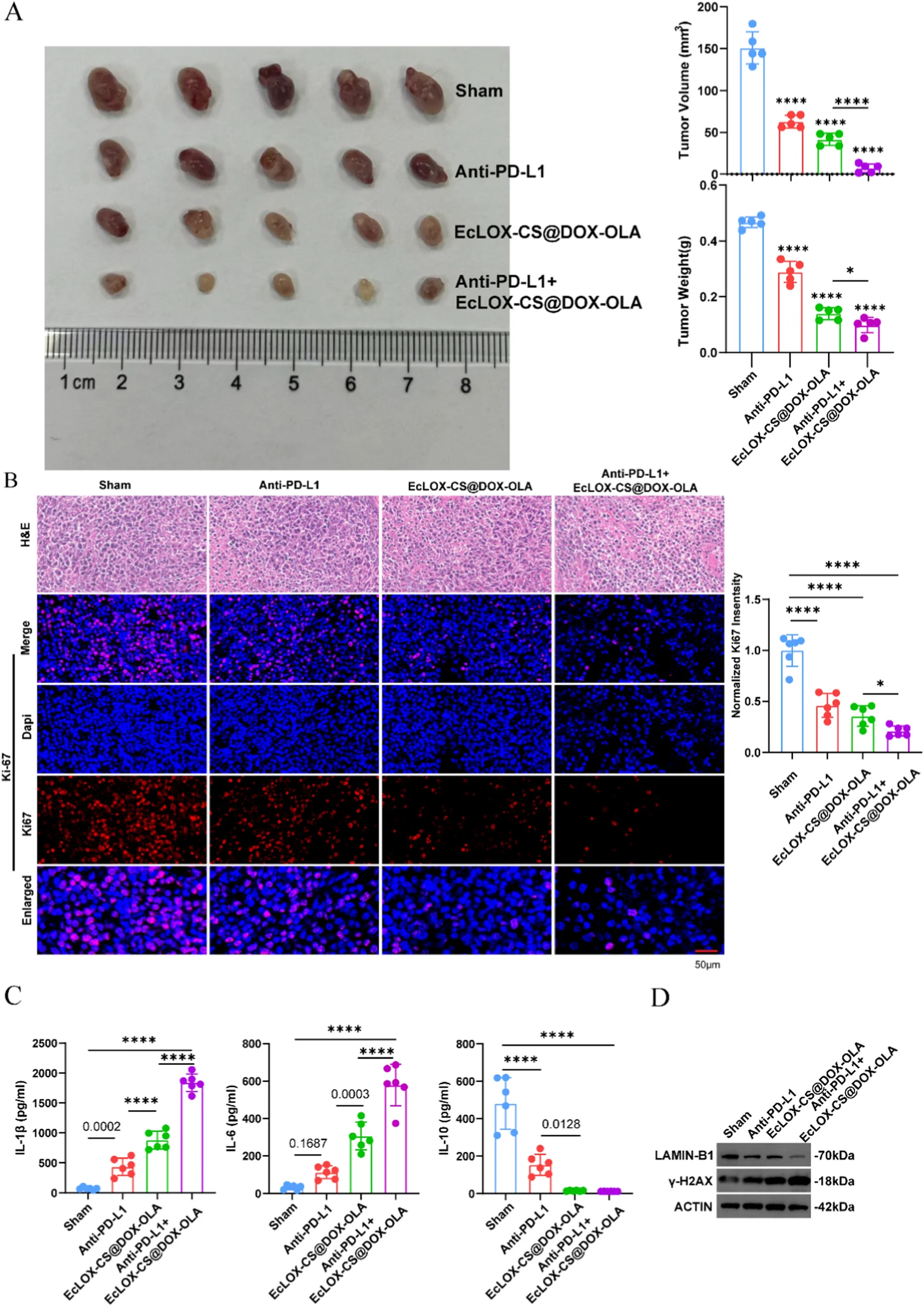
**EcLOX-CS@DOX-OLA synergizes with PD-L1 blockade to enhance antitumor efficacy**. (a) Tumor growth curves, final tumor mass/volume, and representative images from combination therapy (n = 5). (b) Tumor cell proliferation assessed by Ki67 immunohistochemistry (n = 6). (c) Serum cytokine (IL-1β, IL-6, IL-10) levels measured by ELISA (n = 6). (d) Western blot analysis of the DNA damage marker γ-H2AX in tumor lysates. One-way Anova analysis; *p < 0.05, ****p < 0.0001.

Given the robust immune activation, we evaluated the therapeutic potential of combining EcLOX-CS@DOX-OLA with PD-L1 blockade. In mice with orthotopic tumors, monotherapy with either anti-PD-L1 or EcLOX-CS@DOX-OLA alone modestly inhibited tumor growth. However, the combination therapy (EcLOX-CS@DOX-OLA + anti-PD-L1) achieved the most effective outcome, resulting in the smallest tumor size and near-complete suppression of tumor progression (Fig. 7A). Histological analysis provided further evidence of therapeutic efficacy, demonstrating that the combination group exhibited the most substantial inhibition of tumor cell proliferation, as quantified by the lowest Ki67 index (Fig. 7B). Systemic immunological profiling via ELISA revealed that the combination treatment uniquely reshaped the cytokine milieu, significantly elevating pro-inflammatory mediators, while suppressing the immunosuppressive cytokine IL-10 (Fig. 7C). At the molecular level, western blot analysis of tumor lysates confirmed the induction of extensive DNA damage, as indicated by the highest expression of the DNA damage marker γ-H2AX in the EcLOX-CS@DOX-OLA group (Fig. 7D). The combination of EcLOX-CS@DOX-OLA with PD-L1 blockade achieved superior antitumor efficacy, significantly suppressing tumor growth and proliferation, remodeling the immune microenvironment toward a pro-inflammatory state, and enhancing intratumoral DNA damage.

### 2.8 EcLOX-CS@DOX-OLA synergizes with PD-L1 blockade to remodel TME in bladder cancer

**Fig. 8:**
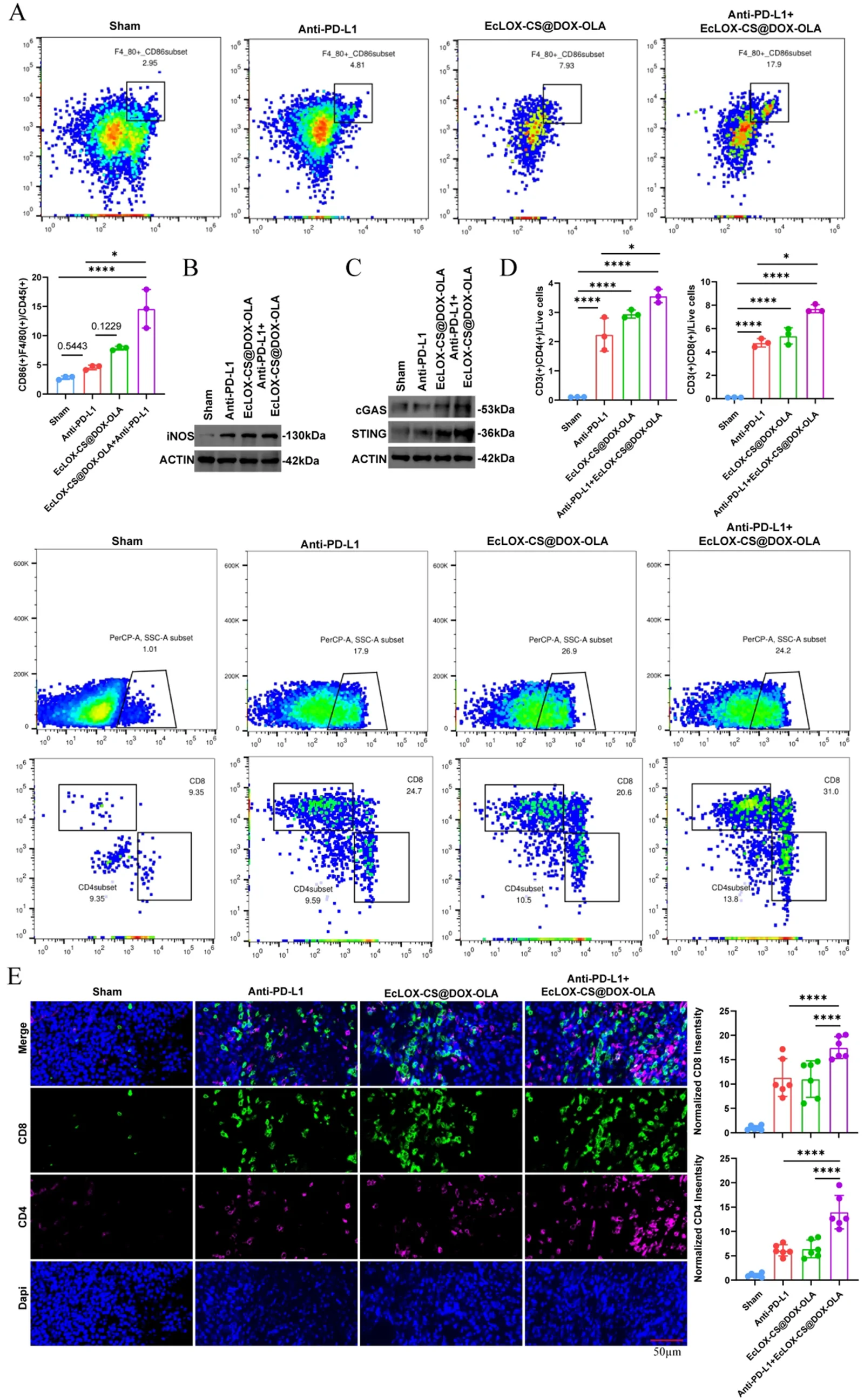
**Combination therapy remodels the tumor immune microenvironment.** (a) Flow cytometric analysis of intratumoral M1 macrophage polarization (n = 3). (b) Western blot analysis of tumor iNOS expression. (c) Western blot analysis of cGAS and STING protein expression in tumors. (d) Flow cytometric analysis of infiltrating CD4^+^ and CD8^+^ T cells (n = 3). (e) IF analysis of CD4^+^ and CD8^+^ T cell infiltration in tumor tissues (n = 6). One-way Anova analysis; *p < 0.05, ****p < 0.0001.

Flow cytometric analysis of tumors from the combination group revealed that combining EcLOX-CS@DOX-OLA with PD-L1 blockade induced the most pronounced polarization of macrophages towards the M1 phenotype (Fig. 8A), which was sustained by WB analysis targeting iNOS (Fig. 8B). Moreover, the combination therapy induced strongest activation of the STING pathway, as indicated by highest up-regulation of both cGAS and STING protein expression (Fig. 8C). Combining EcLOX-CS@DOX-OLA with PD-L1 blockade contributed to the highest levels of infiltrating and activated CD8^+^ and CD4^+^ T cells (Fig. 8D), which was further visualized by IF analysis targeting CD4^+^ and CD8^+^ T cells (Fig. 8E). This demonstrates that the bacteria-chitosan hybrid robot effectively reverses immunosuppression and creates a TME permissive for T cell function, thereby acting synergistically with immune checkpoint blockade to produce a superior and potentially curative therapeutic outcome.

## 3 Methods

### 3.1 Synthesis of chitosan nanoparticles (CS NPs)

Chitosan nanoparticles (CS NPs) were synthesized via the ionic gelation method, based on electrostatic cross-linking between the positively charged amino groups on chitosan chains and the negatively charged phosphate groups of tripolyphosphate (TPP). Briefly, chitosan powder with 95% deacetylation degree and a molecular weight of 200 kDa was dissolved in 1% (v/v) acetic acid to prepare stock solutions at different concentrations. Under continuous magnetic stirring at room temperature, a TPP aqueous solution (0.3 mL, 5 mg mL^−1^) was added dropwise at a constant rate using a syringe pump. Nanoparticles formed spontaneously by adjusting the chitosan-to-TPP mass ratio, which was fixed at 3:1 in this study. The resulting CS NPs were collected by centrifugation, washed with deionized water, and redispersed for further use.

### 3.2 Synthesis of CS@DOX-OLA nanoparticles

To construct a nanodelivery system co-loaded with doxorubicin (DOX) and olaparib (OLA), blank CS NPs were first prepared as described above. Subsequently, under continuous magnetic stirring, DOX solution (1 mg mL^−1^), OLA solution (1 mg mL^−1^), and TPP solution (5 mg mL^−1^) were sequentially added dropwise at a rate of 30 μL min^−1^ using a syringe pump into the CS NP suspension. After complete addition, the mixture was stirred for an additional 30 min at room temperature to allow efficient drug loading via electrostatic and hydrophobic interactions. Finally, the co-loaded CS@DOX-OLA nanoparticles were isolated by centrifugation, washed, and redispersed in PBS (pH 7.4) for storage at 4°C.

### 3.3 Characterization

The surface morphology and dispersion state of different CS nanoparticles were observed by scanning electron microscopy (SEM). Transmission electron microscopy (TEM, EM-200CX, JEOL) was used to examine the microstructure of bacteria and their composites with nanoparticles. The hydrodynamic diameter, polydispersity index (PDI), and zeta potential of the nanoparticles were determined using a ZetaSizer Nano ZS series instrument (Malvern Instruments) via dynamic light scattering. Samples were appropriately diluted with deionized water prior to measurement, and each sample was analyzed in triplicate. The loading efficiency and release concentrations of DOX and OLA were quantified by high-performance liquid chromatography-mass spectrometry (HPLC-MS).

### 3.4 Construction of EcLOX

The EcLOX strain was constructed using *Escherichia coli* Nissle 1917 (Ec) as the chassis. The lactate oxidase gene FG342_RS14265 was amplified from the genomic DNA of Lacticaseibacillus rhamnosus, using KOD One PCR Master Mix (TOYOBO, KMM-201). Meanwhile, the plasmid backbone was amplified from the pUC57 vector. Synthetic long-chain oligonucleotides served as the template to amplify the secretion module, which includes the constitutive promoter J23100 and the pelB secretion signal peptide. These three fragments were assembled via seamless cloning using the MultiF Seamless Assembly Mix (ABclonal). After sequence verification, the constructed plasmid was electroporated into *E. coli* Nissle 1917 to generate the EcLOX strain.

### 3.5 Synthesis of the EcLOX-CS hybrid system

A bacteria-nanoparticle hybrid system was constructed based on electrostatic adsorption. Specifically, 5 mg of CS@DOX-OLA nanoparticles were dispersed in 10 mL of sterile PBS (pH 7.4), followed by the addition of engineered EcLOX bacteria in the logarithmic growth phase (bacterial concentration: 1 × 10^9^ CFU mL^−1^). The mixture was gently shaken and incubated at 37°C for 30 min, allowing the positively charged CS@DOX-OLA nanoparticles to efficiently anchor onto the negatively charged bacterial surface via electrostatic interactions. The hybrid system was then collected by low-speed centrifugation (3000 rpm, 5 min), washed twice with PBS to remove unbound nanoparticles, and finally resuspended in PBS for subsequent experiments.

### 3.6 Stability evaluation of CS@DOX-OLA nanoparticles and EcLOX-CS

The stability of the nano-formulations in a simulated physiological environment was assessed using dynamic light scattering. CS@DOX-OLA nanoparticles and the EcLOX-CS hybrid system were separately dispersed in PBS (pH 7.4) and stored at 4°C for 7 days. Samples were taken at fixed time points each day, and changes in hydrodynamic diameter, PDI, and zeta potential were measured using a particle size-zeta potential analyzer. Meanwhile, the morphological features of the nanoparticles and the stability of the bacteria-nanoparticle composite structure were observed by SEM and TEM.

### 3.7 Drug release study

The drug release behavior of CS@DOX-OLA nanoparticles was investigated using the equilibrium dialysis method. An aliquot of nanoparticle suspension equivalent to 1 mg of DOX was placed in a dialysis bag (molecular weight cutoff: 3.5 kDa) and immersed in PBS release medium (pH 7.4). The system was incubated in a constant-temperature shaker at 37°C (100 rpm). At predetermined time points, release medium was collected and replaced with an equal volume of pre-warmed fresh medium. The concentration of DOX was measured using a microplate reader at an excitation wavelength of 480 nm, while OLA concentration was quantified by HPLC-MS. The cumulative release percentage was calculated based on standard curves, and drug release kinetics were plotted accordingly.

### 3.8 Bacterial viability assessment of EcLOX-CS

The impact of nanoparticle modification on bacterial viability was evaluated by the plate colony counting method. Ec, EcLOX, and EcLOX-CS were adjusted to the same optical density (OD_600_ = 1.0) using PBS. Then, 150 μL of each bacterial suspension was evenly spread on LB agar plates and incubated at 37°C for 24 h. Colony morphology was observed, and colony-forming units (CFU mL^−1^) were counted using an automatic colony counter. The survival rate of bacteria after nano-complexation was evaluated by comparing the CFU mL^−1^ values.

### 3.9 Lactate consumption assay of EcLOX-CS

Ec, EcLOX, and EcLOX-CS were separately inoculated into LB medium containing 20 mM lactate (initial pH 6.5) and incubated statically at 37°C. Culture supernatants were collected at 12 h and 24 h, and the residual lactate concentration in the medium was quantitatively analyzed using a lactate detection kit (based on the LDH enzymatic method). This assay was used to evaluate the lactate-metabolizing capacity of different bacterial strains and the hybrid system.

### 3.10 Cell and murine orthotopic mice model

The MB49 murine bladder cancer cell line (ATCC) was cultured in DMEM high glucose medium supplemented with 10% fetal bovine serum (Gibco) at 37°C with 5% CO_2_. An orthotopic bladder cancer model was established using these cells. Briefly, 1 × 10^6^ MB49 cells suspended in Matrigel (Corning, #354230) were instilled intravesically into mice. Mice were sacrificed on day 14 post-instillation for tumor burden analysis by bladder weight.

### 3.11 WB analysis

Protein lysates were prepared from MB49 cells using RIPA buffer supplemented with protease and phosphatase inhibitors (Thermo Fisher Scientific). Following separation by SDS-PAGE, proteins were transferred to PVDF membranes (Millipore). The membranes were incubated overnight at 4°C with the following primary antibodies (Proteintech): anti-cGAS (26416-1-AP), anti-STING (19851-1-AP), anti-iNOS (22226-1-AP), anti-LAMINB (12987-1-AP), anti-Phospho-Histone H2A.X (83307-2-RR), and anti-ACTIN. Subsequently, membranes were incubated with an appropriate horseradish peroxidase (HRP)-conjugated secondary antibody.

### 3.12 Flow cytometry

For flow cytometry analysis, cells were harvested and resuspended in staining buffer. A total of 2 × 10^6^ cells per 100 μl were first blocked with TruStain FcX (Biolegend, 1:500) for 30 min at 4°C. Subsequently, cells were stained for 30 min at 4°C in the dark with the following fluorophore-conjugated antibodies prepared in staining buffer: anti-CD86 (BD Biosciences, 1:1000), anti-CD45 (BD Biosciences, 1:1000), anti-F4/80 (Biolegend, 1:200), and a viability dye (Live/Dead Aqua, Invitrogen, 1:250). Following staining, cells were fixed in BD Cytofix buffer for 20 min at 4°C, washed, and finally resuspended in FACS buffer (PBS containing 1.5% BSA) for acquisition.

### 3.13 Cell viability

Cell viability was quantified using a Cell Counting Kit-8 (CCK-8; Yeasen) according to the manufacturer's instructions. Briefly, cells were seeded in 96-well plates at a density of 1 × 10^4^ cells per well and allowed to adhere overnight. Following the indicated experimental treatments, 10 μL of CCK-8 reagent was added to each well, and the plates were incubated for 1 h at 37°C. Absorbance was subsequently measured at 450 nm using a Varioskan Flash microplate reader (Thermo Scientific).

### 3.14 Measurement of mitochondrial ROS

Mitochondrial ROS were measured using the Mito-SOX Red fluorescent probe. MB49 cells seeded in 12-well plates were treated for 24 h with 500 μM H_2_O_2_ alone or in combination with the specified formulations. Following treatment, cells were washed with PBS and incubated with 5 μM Mito-SOX Red at 37°C for 10 min. After washing to remove excess probe, cells were immediately imaged using confocal laser scanning microscopy.

### 3.15 Statistical analyses

Statistical analyses were performed using GraphPad Prism software. Data are presented as the mean ± standard deviation (SD). Differences between two experimental groups were assessed using an unpaired, two-tailed Student's *t*-test. For comparisons among three or more groups, one-way analysis of variance (ANOVA) was employed. A p-value of less than 0.05 was considered statistically significant.

## 4 Discussion

From bench to bedside, the discovery that diverse molecular mechanisms—ranging from RNA modification and protein stabilization to tumor microenvironment signaling—converge to enhance DDR underpins treatment resistance in bladder cancer and has catalyzed the development of targeted DDR inhibitors as a cornerstone of precision oncology. The RNA-modifying enzyme NAT10 promotes cisplatin resistance in bladder cancer by enhancing DNA damage repair, by stabilizing AHNAK mRNA, which is required for efficient DNA repair. Consequently, NAT10 is linked to poor patient outcomes [18]. Another protein HNRNPU also regulates cisplatin resistance in bladder cancer by controlling genes involved in DNA damage repair. HNRNPU regulates the expression of the gene NF1, which influences sensitivity to chemotherapy [19]. Cancer-associated fibroblasts (CAFs) promote cisplatin resistance in bladder cancer by secreting CXCL14, which activates the CCR7-STAT3 axis in tumor cells to upregulate the DNA repair gene ERCC4. CCR7-mediated STAT3 activation increases glycolytic metabolism, creating a feedback loop that further activates CAFs and sustains the chemo-resistant environment [20]. The protein YEATS4 promotes cisplatin resistance in bladder cancer, through interfering with DNA damage repair pathways. YEATS4 stability is controlled by KAT8-mediated acetylation, which prevents its degradation and leads to chemoresistance [21]. Reduced expression of the DNA repair protein XPA in bladder cancer is associated with increased chromosomal aberrations. Downregulation of XPA impairs the repair of DNA double-strand breaks, which promotes greater cell viability and genomic instability, facilitating carcinogenesis by allowing the accumulation of mutations in oncogenes and tumor suppressors [22]. The strategy of exploiting DDR deficiencies has moved from a biological concept to a validated clinical approach in bladder cancer, with PARP inhibitors at the forefront. Combining DDR inhibitors with antibody-drug conjugates is an emerging strategy being explored in advanced urothelial carcinoma to overcome treatment resistance and enhance antitumor efficacy [23]. The intricate molecular mechanisms driving cisplatin resistance in bladder cancer, collectively underscore that enhanced DNA damage repair is a central therapeutic challenge. This fundamental understanding has now been translated into the clinical development of targeted DDR inhibitors and their novel combinations, marking a pivotal shift toward precision medicine in overcoming treatment resistance. In our study, the chitosan nanoparticle CS@DOX-OLA was engineered to co-deliver DOX and the PARP inhibitor OLA, creating a synergistic mechanism that exacerbates DNA damage while simultaneously blocking its repair. This dual action not only enhances direct tumor cell killing but also leads to the accumulation of cytoplasmic DNA, which potently activates the cGAS-STING pathway. Consequently, this nanoparticle-mediated disruption of DNA integrity shifts the tumor microenvironment from immunosuppressive to immunogenic, significantly inhibiting cancer progression and synergizing with immune checkpoint blockade for superior therapeutic outcomes.

While cisplatin-based therapy and immunotherapy form the cornerstone of bladder cancer management, treatment resistance in a subset of patients is linked to the overexpression of the aryl hydrocarbon receptor (AhR). The activation of AhR by tryptophan metabolites like kynurenine (Kyn) promotes the ubiquitination and degradation of the STING protein, thereby suppressing interferon production and anti-tumor immunity, which drives chemoresistance; consequently, targeting this axis—through tryptophan restriction or IDO1 inhibition to block Kyn production—represents a promising strategy to restore STING-mediated immune response and overcome therapeutic resistance in bladder cancer [24,25]. The cGAS-STING pathway exerts context-dependent, dual roles in cancer, dictated by three regulatory layers including cell-type specificity. While canonical STING signaling generally promotes anti-tumor immunity by inducing IFN and activating CD8^+^ T cells [26], non-canonical (NC) STING signaling can drive tumor progression—for instance, by upregulating immunosuppressive cytokines like IL-35 in Breg cells to inhibit CD8^+^ T and NK cells [27], activating pro-survival NF-κB/BCL-2 in cancer cells [28], or inducing IL-6 to polarize macrophages toward a pro-tumor M2 phenotype [29]. Therefore, precisely targeting STING modulators based on cellular context is essential to harness its therapeutic potential. In our study, the chitosan nanoparticle CS@DOX-OLA was specifically engineered to activate the STING pathway through a multi-pronged approach. By co-delivering doxorubicin to cause DNA damage and the PARP inhibitor olaparib to block its repair, the nanoparticle ensures the accumulation of cytoplasmic DNA, a potent ligand for cGAS-STING activation, while the chitosan carrier itself further amplifies STING signaling. This concerted STING activation transforms the immunosuppressive tumor microenvironment, significantly inhibiting bladder cancer progression and creating a potent synergy with PD-1/PD-L1 checkpoint blockade therapy.

The profound metabolic reprogramming, characterized by avid glucose consumption and lactate production, not only fuels tumor growth but also actively sculpts an immunosuppressive tumor microenvironment by inhibiting T-cell function, thereby presenting a compelling rationale for therapeutic strategies aimed at disrupting this metabolic-immune axis. Positron emission tomography/computed tomography (PET/CT) imaging with 18F-FDG demonstrates increased glucose uptake in urothelial carcinoma (UC) tumors and metastases [30], a finding consistent with *in vitro* observations that UC cell lines uptake more glucose and produce more pyruvate and lactate than normal cells [31]. Analysis of patient tumor samples, however, reveals significantly lower glucose levels within UC tissue compared to normal urothelium, alongside increases in late TCA cycle intermediates (indicating anaplerotic flux) and the PPP end-product ribose [32]. This collective evidence indicates that UC reprograms its metabolism to rapidly consume glucose, supporting energy production via glycolysis, biomass generation through the PPP and anaplerosis, and redox balance via PPP-derived NADPH [33,34]. Elevated tumor cell metabolism depletes glucose and amino acids in the TME, creating metabolic competition that impairs glycolysis-dependent effector T cell function [[35], [36], [37]]. However, recent evidence indicates T cell viability and effector functions can be preserved under glucose restriction, likely through metabolic adaptation to alternative substrates such as inosine or by replenishing key intermediates like phosphoenolpyruvate [38]. Lactate accumulation in the TME potently suppresses T-cell function, primarily through acidification. Intracellular acidification disrupts cytotoxic T cell metabolism [39,40], with lactic acid (low pH) impairing CD8^+^ T cell cytotoxicity, while sodium lactate (neutral pH) alters CD4^+^ T helper cell function [41]. Notably, neutralizing this tumor acidity—achieved in preclinical models with oral bicarbonate, which increased intratumoral pH from 6.8 to 7.0 and synergized with immunotherapies like anti-PD-1 [42]—presents a feasible strategy, as equivalent bicarbonate doses have been well-tolerated in human safety studies [43]. The Warburg effect drives rapid glucose consumption and substantial lactate production, creating an acidic TME that potently suppresses anti-tumor T cell function. Consequently, inhibiting lactate accumulation or neutralizing its associated acidity represents a promising therapeutic strategy to reverse this immunosuppression and potentially improve responses to immunotherapy. In our study, the nanoparticle system, when assembled with lactate-oxidase-expressing bacteria into the EcLOX-CS@DOX-OLA hybrid robot, directly targets the acidic TME by continuously consuming immunosuppressive lactate. This metabolic normalization reverses acidity-driven immunosuppression, which enhances the efficacy of the DOX and DNA repair inhibitor. By remodeling the acidic TME, the nanoparticle-bacteria hybrid cripples a key promoter of cancer progression and synergistically improves tumor control.

## 5 Conclusion

This study constructs an engineered bacteria-chitosan hybrid robot, EcLOX-CS@DOX-OLA, that actively targets tumors, depletes immunosuppressive lactate, and synergistically activates the cGAS-STING pathway through co-delivered doxorubicin and olaparib. EcLOX-CS@DOX-OLA potently remodels the tumor immune microenvironment and significantly enhances the efficacy of immune checkpoint blockade therapy for bladder cancer.

## CRediT authorship contribution statement

**Baodan Zhang:** Writing – original draft. **Jie Zhu:** Writing – original draft. **Zhenyu Hong:** Data curation. **Yuhang Wang:** Data curation. **Yunong Wang:** Investigation. **Fangdie Ye:** Formal analysis. **Liping Tao:** Supervision, Project administration. **Yongyong Lu:** Supervision, Project administration.

## Ethics approval and consent to participate

Not applicable.

## Availability of data and material

Data available within the article or its supplementary materials.

## Consent for publication

Not applicable.

## Clinical trial number

Not applicable.

## Funding

This work was supported by a grant from the Basic Scientific Research Programs of wenzhou (No. Y20210178).

## Declaration of competing interests

The authors declare that they have no known competing financial interests or personal relationships that could have appeared to influence the work reported in this paper.

## Data Availability

Data will be made available on request.
