# Supplementary material for: An engineered bacteria-chitosan hybrid robot for lactate depletion and synergistic STING activation to potentiate bladder cancer immunotherapy
Source: Mater Today Bio. 2026 Aug 25;40:103593. doi: 10.1016/j.mtbio.2026.103593 (PMC13563556; doi:10.1016/j.mtbio.2026.103593)
Supplement: Supplementary file 1 [file mmc1.docx]

**Figure S1.** TEM images of CS nanoparticles.

**Figure S2.** PDI stability of CS@DOX-OLA nanoparticles (n = 3).

**Figure S3.** Particle size and zeta potential stability of CS@DOX-OLA nanoparticles in urine medium (n = 3).

**Figure S4.** DOX release profiles of CS@DOX OLA nanoparticles under pH 6.5 release medium (n = 3).

**Figure S5.** DOX (left) and OLA (right) loading efficiency of CS@DOX-OLA nanoparticles after one-week storage (n = 3).

**Figure S6.** Cumulative release of OLA of CS@DOX-OLA nanoparticles (n = 3).

**Figure S7.** (A) Stability of particle size of EcLOX@CS in the urine medium (n = 3). (B) Stability of zeta potential of EcLOX@CS in the urine medium. (C) bacterial colony count of EcLOX@CS in PBS (left) and urine medium (right) (n = 3). (D) DOX release of EcLOX@CS in the urine medium after 12 h incubation (n = 3). One-way Anova analysis; ns, no significant

**Figure S8.** Colony photographs of different bacteria onto LB agar plates after one-week storage.

**Figure S9.** Relative lactate consumption in a lactate-added medium after treatment with different bacteria after one-week storage (n = 3). One-way Anova analysis.

**Figure S10.** Western blot analysis of cGAS-STING pathway activation.

Representative Western blot images of cGAS, STING, and β‑Actin in cells treated with different formulations.

**Figure S11.** The activation of nano-particle-mediated ICD activation in the treated tumor cells in vitro by assessing ATP and HGMB levels, n=6. One-way Anova analysis; **** p < 0.0001.

**Figure S12.** The systematic review the in vivo safety of EcLOX-CS@DOX-OLA. (A) The H&E staining of various major organs in the mice treated or not treated with EcLOX-CS@DOX-OLA. (B) The secretion of inflammatory cytokines in the peripheral blood of mice treated or not treated with EcLOX-CS@DOX-OLA using ELISA analysis, n=6. Two-tailed Student's t-test; ns, no significant.

**Figure S13.** The secretion of inflammatory cytokines in the treated bladder tissue in vivo using ELISA analysis, n=6. One-way Anova analysis; ns, no significant.
